# Nurses’ Attitudes Toward the Importance of Families in Nursing Care: A Multinational Comparative Study

**DOI:** 10.1177/10748407211042338

**Published:** 2021-09-08

**Authors:** Lisa A. Cranley, Simon Ching Lam, Sarah Brennenstuhl, Zarina Nahar Kabir, Anne-Marie Boström, Angela Yee Man Leung, Hanne Konradsen

**Affiliations:** 1 University of Toronto, Ontario, Canada; 2 The Hong Kong Polytechnic University, Kowloon, Hong Kong; 3 Karolinska Institutet, Stockholm, Sweden; 4 Karolinska University Hospital, Stockholm, Sweden; 5 Stockholms Sjukhem, Sweden; 6 University of Alberta, Edmonton, Canada; 7 University of Copenhagen, Denmark; 8 Herlev and Gentofte Hospital, Denmark

**Keywords:** nurse attitudes, family-focused care, survey, cross-sectional, cross-national comparisons

## Abstract

The aim of this study was to examine nurses’ attitudes about the importance of family in nursing care from an international perspective. We used a cross-sectional design. Data were collected online using the Families’ Importance in Nursing Care—Nurses’ Attitudes (FINC-NA) questionnaire from a convenience sample of 740 registered nurses across health care sectors from Sweden, Ontario, Canada, and Hong Kong, China. Mean levels of attitudes were compared across countries using analysis of variance (ANOVA). Multiple regression was used to identify factors associated with nurses’ attitudes and to test for interactions by country. Factors associated with nurse attitudes included country, age, gender, and several practice areas. On average, nurses working in Hong Kong had less positive attitudes compared with Canada and Sweden. The effects of predictors on nurses’ attitudes did not vary by country. Knowledge of nurses’ attitudes could lead to the development of tailored interventions that facilitate nurse-family partnerships in care.

## Background

Family involvement in care processes is an important part of providing patient- and family-focused care and ensuring optimal patient outcomes (Mackie et al., 2018; Park & Schumacher, 2014; Petriwskyj et al., 2014). Nurses play a central role in advocating and facilitating patient- and family-focused care practices (Mackie et al., 2018), including social support for high family function and health (Shamali et al., 2019). Family is a broad term that includes relatives, friends, neighbors, or other individuals significant to the patient (Benzein, Johansson, Arestedt, & Saveman, 2008). From a Family Systems Nursing perspective, family is conceptualized as the interaction, reciprocity, and relationships between multiple systems (e.g., patient, family, nurse, health care system; Bell, 2009). Family is the unit of care (Bell, 2009; Wright & Leahey, 1990) and family members’ involvement as care partners is key to providing quality patient care (Astedt-Kurki et al., 2001; Saveman et al., 2011; Voltelen et al., 2016). Family might be involved in various supporting roles, such as accompanying the patient to health care appointments and procedures, providing emotional support, care provision (Gusdal et al., 2017; Luttik et al., 2007), and surrogate (proxy) decision-making (Petriwskyj et al., 2014). Despite its importance, family involvement in care can be a challenging and complex process for health care providers and family (Petriwskyj et al., 2014). For example, lack of education about how to conduct therapeutic conversations with families and lack of time can act as barriers to nurses actively involving families in care (Hoplock et al., 2019; Saveman, 2010).

A recent integrative review found that nurses’ attitudes toward families may also help or hinder family’s involvement in care (Mackie et al., 2018). While positive attitudes toward families can lead to better communication, relationships, and outcomes (Hoplock et al., 2019; Saveman, 2010), less supportive attitudes may result in negative feelings among family members (e.g., feeling excluded and less empowered to participate in care; Hoplock et al., 2019). From a reasoned action perspective, an attitude is defined as an individual’s evaluation of an object, concept, or behavior based on the degree of favorableness or unfavorableness (Ajzen & Fishbein, 2000). Attitudes are determined by an individual’s beliefs and can guide one’s behavior (Ajzen & Fishbein, 2000). Importantly, attitudes can also change (Ajzen & Fishbein, 2000). As highlighted by Hoplock and colleagues (2019), understanding nurses’ attitudes toward family importance in nursing care is of critical importance as attitudes can affect both nurses’ and family members’ behavior. Families are an important part of care planning and delivery (Saveman, 2010), and nurses’ attitudes toward the importance of family in the care process can contribute to the quality of the relationship that develops between nurses and family (Alfaro Diaz et al., 2019). Linnarsson and colleagues (2014) reported that a positive attitude toward patients’ families was associated with actively involving family members in care.

Research examining nurses’ attitudes toward families’ importance in nursing care has found that in general, nurses have positive attitudes (Benzein, Johansson, Arestedt, & Saveman, 2008; Blondal et al., 2014; Gusdal et al., 2017; Hoplock et al., 2019; Hsiao & Tsai, 2015; Linnarsson et al., 2014; Luttik et al., 2017; Østergaard et al., 2020; Sveinbjarnardottir et al., 2011). Recent literature provides some evidence that nurses’ attitudes vary based on individual characteristics. In general, nurses who expressed more positive attitudes toward family importance in nursing care are older (Blondal et al., 2014; Østergaard et al., 2020); female (Linnarsson et al., 2014; Sveinbjarnardottir et al., 2011); have higher education levels (e.g., master’s or doctorate degree; Hagedoorn et al., 2020; Luttik et al., 2017; Østergaard et al., 2020); have more clinical experience (e.g., more than 7 years’ experience; Blondal et al., 2014; Hagedoorn et al., 2020; Østergaard et al., 2020); have had a seriously ill family member in need of professional care (Benzein, Johansson, Arestedt, & Saveman, 2008; Hsiao & Tsai, 2015; Linnarsson et al., 2014; Østergaard et al., 2020; Sveinbjarnardottir et al., 2011); and are employed in a workplace with a general approach to the care of families (i.e., the health care organization has a general philosophy in place about the care of families; Benzein, Johansson, Arestedt, & Saveman, 2008; Gusdal et al., 2017; Hoplock et al., 2019).

Nurses’ attitudes toward involving families in care have been studied in various health care settings and/or specialties (Benzein, Johansson, Arestedt, & Saveman, 2008), for example, hospital/acute care (Blondal et al., 2014; Linnarsson et al., 2014); primary care (Oliveira et al., 2011); cardiovascular care (Gusdal et al., 2017; Luttik et al., 2017); psychiatric/mental health care (Hsiao & Tsai, 2015; Sveinbjarnardottir et al., 2011); and pediatric care (Oh et al., 2018). Studies including more than one country (Luttik et al., 2017) or more than one health care setting in their sample have reported variation in nurses’ attitudes toward family importance in care (Benzein, Johansson, Arestedt, & Saveman, 2008; Gusdal et al., 2017; Hagedoorn et al., 2020; Hsiao & Tsai, 2015; Luttik et al., 2017; Østergaard et al., 2020). Luttik et al. (2017) found that nurses living in Scandinavia had more positive attitudes than nurses working in Belgium. Studies have also reported that hospital nurses had less supportive attitudes than nurses in primary health care (Benzein, Johansson, Arestedt, & Saveman, 2008; Gusdal et al., 2017; Hagedoorn et al., 2020; Østergaard et al., 2020) or home care (Hagedoorn et al., 2020). However, in a Canadian study, Hoplock et al. (2019) found no statistically significant differences in attitudes between hospital nurses and home visiting nurses.

While there has been a growing body of literature on nurses’ attitudes toward family importance in care, direct comparisons and interpretation across studies are difficult due to differences in the inclusion criteria, sampling method, and demographic data collected. Such discrepancies among these studies hinder a comprehensive understanding of nurses’ attitudes about family importance in nursing care. This study seeks to address this gap—this the first study to our knowledge to examine nurses’ attitudes toward the importance of family in nursing care across all health care sectors from three countries. Few studies have examined nurses’ attitudes toward families’ importance in care from an international perspective (Luttik et al., 2017). There have been increasing calls for greater cross-national comparative studies on nurse attitudes toward the importance of family in care for a more comprehensive understanding of country similarities and differences (Gusdal et al., 2017; Hoplock et al., 2019; Luttik et al., 2017; Østergaard et al., 2020). Knowledge of nurses’ attitudes of family importance in nursing care could lead to the development of education programs or interventions that facilitate collaboration and partnerships in care, implementation of policies, or organizational changes to involve families in care (Benzein, Johansson, Arestedt, & Saveman, 2008; Hoplock et al., 2019; Sveinbjarnardottir et al., 2011; Yamazaki et al., 2017).

The aim of this study was to examine nurses’ attitudes about the importance of family in nursing care from an international perspective. The specific study objectives were to (1) describe and compare the level of nurse attitudes of the importance of family in nursing care across three countries; (2a) identify predictors of nurse attitudes toward family importance in nursing care; and (2b) determine whether predictors vary by country.

## Method

### Design and Sample

A cross-sectional study design was used to guide the study. The Strengthening the Reporting of Observational Studies in Epidemiology (STROBE) checklist for cross-sectional studies is appended (von Elm et al., 2007; Supplemental Appendix S1). Data were collected from a convenience sample of registered nurses across health care sectors from Sweden, Ontario, Canada, and Hong Kong Special Administrative Region of the People’s Republic of China (hereafter Hong Kong, China). The three countries were purposefully selected based on our research team’s geographic locations. Registered nurses currently working in any health care setting (e.g., hospital, home and community care, long-term care) were eligible to participate. Excluded were student nurses, registered nurses not currently employed or retired, nursing assistants, registered/licensed practical nurses, and nurse practitioners.

An invitation to complete the online questionnaire was sent to nursing associations or other professional nursing interest groups and through social media (e.g., professional Facebook groups, Twitter, WhatsApp). The invitation letter had information about the study purpose, voluntary participation, confidentiality of the data, and implied consent from those who complete and submit the survey. The invitation included a request for respondents to complete the survey only once. Snowball sampling was also used as a recruitment strategy, by asking respondents to recruit additional nurses (Patton, 2014). The invitation letter included a request for respondents to send the invitation and link to the questionnaire to other registered nurses they knew who were currently working, through Facebook contacts or other electronic means (Sadler et al., 2010). A target sample size was estimated using rules of thumb for descriptive research based on population size because effect sizes for differences in the countries selected were unknown (Hill, 1998). A sample of 385 per country was selected as returns in power for increased sample size begin to plateau at this number (Hill, 1998).

### Ethical Considerations

Ethics approval was obtained from the University of Toronto, Ontario, Canada (Reference #37882), the Hong Kong Polytechnic University’s Research Ethics Board (Reference #: HSEARS20190520001), and the Swedish Regional Board of Ethics (Reference 2018/1535-31). The survey was voluntary, confidential, and anonymous.

### Data Collection

Questionnaires were distributed online via a survey link provided in the study invitation letter. Some advantages to using an online survey to collect data include ease of implementation, reduced costs, respondents can answer the questions at their own convenience, and it has potential to increase response rates (Dillman et al., 2009). Data were collected in Sweden from October 2018 to March 2019, in Hong Kong, China, from June 2019 to March 2020, and in Ontario, Canada, from July 2019 to March 2020. We sent online reminders to complete the survey to nursing associations and other professional nursing groups and through social media (depending on the country; Dillman et al., 2009).

### Measures

#### Demographic characteristics

Demographic variables included in the survey were nurses’ age, gender, workplace setting, clinical specialty, education level, and have had a seriously ill family member in need of professional care. As educational requirements for nursing licensure vary across the countries examined, we assessed the education level according to whether the nurse had obtained the first obligatory professional training in nursing (bachelors or diploma in Ontario, Canada, and Hong Kong, China, and bachelors in Sweden) or had obtained a higher level of education (e.g., postgraduate specialization certificate, masters, PhD). In total, eight practice areas were derived based on clinical specialty and workplace setting and included the following: (a) pediatric care (e.g., pediatric emergency, pediatric oncology); (b) maternal care (e.g., obstetrics, neonatal care); (c) geriatric care (e.g., gerontology, long-term care); (d) general medicine/surgery, herein called medical-surgical (e.g., oncology, cardiology, surgery, operating theater); (e) critical/acute care (e.g., intensive care, emergency department); (f) mental health care (e.g., psychiatry); (g) any direct care provided outside of hospitals (e.g., public health, primary care, home care, community nursing); and (h) nondirect care (e.g., care coordination, research, education, administration).

#### Families’ Importance in Nursing Care—Nurses’ Attitudes (FINC-NA) Questionnaire

The FINC-NA questionnaire was the outcome measure. FINC-NA is a 26-item questionnaire originally developed in Sweden by Benzein and colleagues that measures the attitudes of nurses toward the importance of involving families in nursing care (Benzein, Johansson, Arestedt, Berg, & Saveman, 2008). The FINC-NA questionnaire has four subscales: family as a resource in nursing care (Fam-RNC) with 10 items, score range 10 to 50, assesses positive attitudes toward family members and the value of their presence in nursing care (e.g., “Family members should be invited to actively take part in the patient’s nursing care”); family as a conversational partner (Fam-CP) with eight items, score range 8 to 40, assesses attitudes toward the importance of acknowledging the patient’s family members and having dialogue with them (e.g., “I ask family members to take part in discussions from the very first contact, when a patient comes into my care”); family as a burden (Fam-B) with four items, score range 4 to 20, assesses negative attitudes toward the presence family members and time to take care of families (e.g., “The presence of family members makes me feel that they are checking up on me”); and family as its own resource (Fam-OR) with four items, score range 4 to 20, assesses attitudes toward family members as having their own resources for coping (e.g., “I consider family members as co-operating partners”; Benzein, Johansson, Arestedt, Berg, & Saveman, 2008).

In this study, the revised version of the FINC-NA questionnaire was used (Saveman et al., 2011). The English version of the survey was used in Ontario, Canada, and Hong Kong, China, and the Swedish version of the survey was used in Sweden. The Swedish version of the FINC-NA has been validated with Swedish nurses (Benzein, Johansson, Arestedt, Berg, & Saveman, 2008; Saveman et al., 2011). The English version has been used in Canada showing good internal consistency of the scales (Hoplock et al., 2019). In a recent review, the revised FINC-NA was found to be one of the best suited questionnaires to measure the importance of family involvement in clinical practice (Alfaro Diaz et al., 2019). We referred to “family” as individuals considered significant for the patient, such as family members, friends, or neighbors (Benzein, Johansson, Arestedt, & Saveman, 2008). In their refinement of the scale, Saveman and colleagues (2011) revised the item responses to a 5-point Likert-type scale (ranging from 1 = *strongly disagree* to 5 = *strongly agree*), replacing the original 4-point Likert-type scale (Benzein, Johansson, Arestedt, Berg, & Saveman, 2008). Item scores are summed to create a total score that range from 26 to 130. After reverse coding negatively worded items from the Fam-B subscale, higher scores indicated more positive attitudes (i.e., perceived family as less of a burden). The revised FINC-NA has good internal consistency with Cronbach’s alpha .92 for the total scale and greater than .70 for the subscales (range: .72–.86; Saveman et al., 2011). In this study, the internal consistency using Cronbach’s alpha for the total scale ranged from .92 to .94 and the subscales ranged from .70 to .89 (with the exception of the Fam-B subscale for Hong Kong, China, α = .62).

### Data Analysis

The demographic characteristics of the sample were described using summary statistics, such as means and standard deviations for continuous variables (e.g., age) and frequency counts and percentages for nominal variables (e.g., gender). Characteristics were compared across the three countries using analysis of variance (ANOVA) and chi-square tests, as appropriate.

Scores for the FINC-NA were calculated for the overall scale and each subscale. Means and standard deviations were used to describe the level of attitudes in each country. Means were compared using ANOVA followed by pairwise comparisons. Post hoc analyses were conducted using the Bonferroni method with adjusted *p* values. This method of adjusting for multiple comparisons was selected due to the small number of tests required. There were very little item-level missing data (maximum/per item *n* = 3). When item-level missing data occurred, individual-level mean imputation was used.

Multiple linear regression was used to identify predictors of the overall scale and each subscale. Predictors selected a priori included country, age, gender, education, have had a seriously ill family member in need of professional care, and practice area. Reference groups were selected based on the largest sample size to maximize power of comparisons. Model diagnostics were undertaken prior to establishing a final model, including tests of multicollinearity and assessing linearity of the relationship between age and the outcome. Model fit was assessed using the *F* statistic and the adjusted *R*^2^. To determine whether predictors varied according to country, interaction terms were created by multiplying dummy variables representing the three countries by each of the predictors. A model containing main effects only was compared with a second model containing the main effects and interaction terms. An omnibus test of interaction was undertaken testing all interaction terms at once using *F* change statistic for each outcome of interest by comparing the first model with the second. Nonsignificant interactions were removed from the final model. Complete case analysis was used because there were less than 5% missing data in the final multivariable models and no reason to assume the data were not missing at random. The analysis was undertaken using SPSS (V 26).

## Results

### Sample Characteristics

After removing a total of 10 cases with significant missing data (only a few questions were answered) or that did not meet the inclusion criteria (e.g., retired, nurse practitioner), our final sample included a total of 740 nurses, with 164 from Ontario, Canada, 214 from Hong Kong, China, and 362 from Sweden. The mean age ranged from 37 years in Hong Kong, China (*SD* = 10.9), 41 years in Ontario, Canada (*SD* = 13.2), and 42 years in Sweden (*SD* = 9.9). While most nurses in each country were females, the proportion of males varied significantly across countries, from a low of 3.7% in Ontario, Canada, to a high of 20% in Hong Kong, China (*p* < .001). At least a third of the sample from each country had more than the first professional training requirement in nursing (e.g., postgraduate specialization certificate, masters, PhD) with the greatest number coming from Sweden (42%), although the proportion did not vary significantly across countries. More than 80% of nurses from Ontario, Canada, and Sweden have had a seriously ill family member in need of professional care; this proportion was significantly higher than that found in Hong Kong, China (57%; *p* < .001). Although the largest proportion of nurses from each country worked in a hospital medical-surgical unit, some variation in the distribution of practice areas was observed. For example, a higher proportion of nurses worked in maternal care or critical/acute care in Ontario, Canada, than in the other countries, whereas more nurses worked in medical-surgical units in Hong Kong, China, and Sweden than in Ontario, Canada (see Table 1).

**Table 1.** Demographics of the Participants.

Demographic	Ontario, Canada (*n* = 164)	Hong Kong, China (*n* = 214)	Sweden (*n* = 362)	Statistic^a^	*p* value
	*n* (%)	*n* (%)	*n* (%)		
Age—*M* (*SD*)	40.9 (13.2)	36.6 (10.9)	42.1 (9.9)	17.25	<.001
Missing (*n*)	8	2	0		
Gender					
Female	155 (96.3)	156 (80.0)	338 (93.4)	34.35	<.001
Male	6 (3.7)	39 (20.0)	24 (6.6)		
Missing (*n*)	3	19	0		
Education					
First diploma or degree^b^	107 (66.5)	129 (60.3)	207 (57.8)	3.50	.176
Postgraduate education^c^	54 (33.5)	85 (39.7)	151 (42.2)		
Missing (*n*)	3	0	4		
Practice area					
Primary care/home/community care	26 (16.1)	26 (12.9)	37 (10.2)	51.09	<.001
Critical care	34 (21.1)	16 (8.0)	54 (14.9)		
Geriatric care	17 (10.6)	22 (10.9)	43 (11.9)		
Maternal care	18 (11.2)	11 (5.5)	12 (3.3)		
Medical-surgical	33 (20.5)	85 (42.3)	109 (30.1)		
Mental health care	9 (5.6)	17 (8.5)	25 (6.9)		
Pediatric care	10 (6.2)	11 (5.5)	33 (9.1)		
Nondirect care	14 (8.7)	13 (6.5)	49 (13.5)		
Missing (*n*)	3	13	0		
Seriously ill family member^d^					
Yes	137 (83.5)	122 (57.0)	300 (82.9)	56.00	<.001
No	27 (16.5)	92 (43.0)	62 (17.1)		
Missing (*n*)	0	0	0		

a Chi-square test for categorical variables and *F* test for continuous variables. ^b^ First obligatory nursing diploma or degree. ^c^ Postgraduate education (e.g., masters, PhD, postgraduate specialization certificate). ^d^ Have had a seriously ill family member in need of professional care.

### Objective 1. Cross-Country Differences in Nurses’ Attitudes Toward Families’ Importance in Nursing Care

The mean levels of the FINC-NA total scale and subscales for each country are found in Table 2. The total score was significantly lower in Hong Kong, China (*M* = 97.0, *SD* = 11.7), than Ontario, Canada (*M* = 102.1, *SD* = 14.0; *p* = .001), or Sweden (*M* = 104.6, *SD* = 14.8; *p* <.001); however, no significant difference was found between Ontario, Canada, and Sweden (*p* = .171). Analysis of the subscale scores showed significant cross-country differences for Fam-B and Fam-CP subscales. The mean score for Fam-B was highest (perceived family as less of a burden) in Sweden (*M* = 16.3, *SD* = 3.3), followed by Ontario, Canada (*M* = 14.3, *SD* = 3.6), and lowest in Hong Kong, China (*M* = 11.2, *SD* = 2.6), with significant differences being found between each pair of countries (*p* < .001). The mean score for Fam-CP was significantly lower in Hong Kong, China (*M* = 31.3, *SD* = 4.3), than in Ontario, Canada (*M* = 32.8, *SD* = 4.7; *p* = .01), or Sweden (*M* = 33.1, *SD* = 5.3; *p* <.001); however, no significant differences were found between Ontario, Canada, and Sweden (*p* = 1.00).

**Table 2.** Comparison of the Mean Score of the Families’ Importance in Nursing Care—Nurses’ Attitudes (FINC-NA) Questionnaire Using ANOVA Analysis.

Subscale	Ontario, Canada (*n* = 164)	Hong Kong, China (*n* = 214)	Sweden (*n* = 362)	Omnibus *F* test statistic	*p* value
	*M* (*SD*)	*M* (*SD*)	*M* (*SD*)		
Family as a resource in nursing care	39.4 (6.1)	38.9 (5.3)	39.5 (5.5)	0.67	.510
Family as a conversational partner	32.8 (4.7)a	31.3 (4.3)ab	33.1 (5.3)b	9.82	<.001
Family as a burden	14.3 (3.6)ac	11.2 (2.6)ab	16.3 (3.3)bc	169.71	<.001
Family as its own resource	15.7 (2.6)	15.6 (2.4)	15.8 (3.1)	0.15	.865
Total FINC-NA scale	102.1 (14.9)a	97.0 (11.7)ab	104.6 (14.8)b	19.94	<.001

*Note*. Fam-B = reverse scores. Pairwise differences are indicated with matching letters (e.g., within each row, the number with an “a” is significantly different from the number with a matching “a” in the same row); for example, the Fam-B row shows the mean for this subscale in Canada (14.3) is significantly different from the Fam-B mean for Hong Kong (11.2). ANOVA = analysis of variance. Fam-B = family as a burden.

### Objective 2a. Factors Associated With Nurses’ Attitudes Toward Families’ Importance in Nursing Care

Prior to establishing the factors associated with nurses’ attitudes, the presence of interactions by country was tested to ensure correct model specification. There was no evidence that incorporating interactions between each of the factors and country into the model improved fit for the total scale or any of the subscales, judged by nonsignificant *F* change values, *F* change (22, 668) = 0.12–1.3, *p* = .14–.59. As a result, the interactions were removed from the model and Objective 2a was evaluated using main effects only.

The final model of predictors of nurses’ attitudes toward family importance in nursing care is shown in Table 3. For the total scale score, significant predictors of nurse attitudes included country, age, gender, and several practice areas, including critical care, geriatric care, maternal care, pediatric care, and nondirect care. After accounting for all other variables in the model, scores in Hong Kong, China, were significantly lower than those in Sweden (*B* = −4.7, 95% confidence interval [CI] = [−7.2, −2.3]; *p* ≤ .001). Males had significantly lower scores (*B* = −3.6, 95% CI = [−7.0, −0.2]; *p* = .04), while age was associated with higher scores (*B* = 0.3, 95% CI = [0.2, 0.4]; *p* ≤ .001). Finally,

**Table 3.** Predictors of Nurse Attitudes Toward Families’ Importance in Nursing Care—Total Score.

Predictor	*B*	95% CI Lower bound	95% CI Upper bound	*p* value
Ontario, Canada^a^	−2.187	−4.704	0.330	.088
Hong Kong, China	−4.739	−7.223	−2.256	<.001
Education^b^	1.981	−0.117	4.078	.064
Gender^c^	−3.607	−7.049	−0.165	.040
Age	0.321	0.228	0.413	<.001
Seriously ill family^d^	−1.010	−1.373	3.393	.405
Primary care^e^	2.382	−0.877	5.642	.152
Critical care	−4.011	−7.187	−0.834	.013
Geriatric care	4.605	1.272	7.938	.007
Maternal care	5.548	1.058	10.038	.016
Mental health care	3.376	−0.709	7.462	.105
Pediatric care	5.218	1.631	8.806	.004
Nondirect care	9.607	5.630	13.583	<.001
*F* value	13.13 (13, 690)			<.001
Adjusted *r*^2^	.18			

*Note. N* = 704. The total score of Families’ Importance in Nursing Care—Nurses’ Attitudes was used as the dependent variable. Nonsignificant interactions between country and predictors were removed. CI = confidence interval.

a Country reference group = Sweden. ^b^Education reference group = first obligatory nursing diploma or degree. ^c^Gender reference group = female. ^d^Have had a seriously ill family member in need of professional care. ^e^Primary care/home/community care; practice area reference group = medical-surgical unit.

various practice areas were found to have higher or lower scores than the reference group of medical-surgical area. Nurses working in critical care had lower total scores (less positive attitudes) on average (*B* = −4.0, 95% CI = [−7.2, −0.8]; *p* = .01), whereas those working in geriatric care, maternal care, pediatrics, and areas involving nondirect care (e.g., care coordination, academia) had significantly higher scores than nurses working in medical-surgical areas. Taken together, the predictors accounted for 18% of the variation in the total score of FINC-NA (Table 4).

**Table 4.** Predictors of Nurse Attitudes Toward Families’ Importance in Nursing Care—Subscale Scores.

Predictor	Fam-RNC					Fam-CP		
	*B*	95% CI Lower bound	95% CI Upper bound	*p* value	*B*	95% CI lower bound	95% CI upper bound	*p* value
Ontario, Canada^a^	−0.015	−1.047	1.018	.978	−0.258	−1.149	0.632	.569
Hong Kong, China	0.377	−0.641	1.396	.467	−0.760	−1.639	0.118	.090
Education^b^	0.616	−0.244	1.476	.160	0.631	−0.111	1.373	.095
Gender^c^	−1.317	−2.729	0.094	.067	−1.848	−3.065	−0.631	.003
Age	0.096	0.058	0.134	<.001	0.099	0.066	0.132	<.001
Seriously ill family^d^	−0.450	−0.527	1.428	.366	−0.351	−0.492	1.193	.414
Primary care^e^	1.107	−0.229	2.444	.104	0.751	−0.402	1.904	.201
Critical care	−1.629	−2.931	−0.326	.014	−1.075	−2.198	0.049	.061
Geriatric care	1.827	0.461	3.194	.009	1.817	0.638	2.996	.003
Maternal care	1.937	0.096	3.778	.039	1.817	0.229	3.406	.025
Mental health care	0.816	−0.860	2.491	.339	1.790	0.345	3.235	.015
Pediatric care	1.448	−0.023	2.919	.054	1.850	0.581	3.119	.004
Nondirect care	3.706	2.075	5.336	<.001	3.087	1.680	4.493	<.001
*F* value	6.82 (13, 690)			<.001	10.06 (13, 690)			<.001
Adjusted *r*^2^	.10				.14			
Predictor	Fam-B					Fam-OR		
	*B*	95% CI Lower bound	95% CI upper bound	*p* value	*B*	95% CI Lower bound	95% CI upper bound	*p* value
Ontario, Canada^a^	−1.840	−2.429	−1.251	<.001	−0.064	−0.586	0.457	.808
Hong Kong, China	−4.630	−5.212	−4.049	<.001	0.275	−0.240	0.790	.294
Education^b^	0.327	−0.164	0.818	.191	0.410	−0.025	0.844	.065
Gender^c^	0.017	−0.789	0.822	.967	−0.459	−1.172	0.255	.207
Age	0.081	0.059	0.103	<.001	0.045	0.025	0.064	<.001
Seriously ill family^d^	−0.217	−0.341	0.775	.446	0	−0.495	0.457	1.000
Primary care^e^	−0.045	−0.808	0.718	.907	0.573	−0.103	1.249	.096
Critical care	−0.873	−1.616	−0.129	.021	−0.434	−1.093	0.224	.196
Geriatric care	0.476	−0.304	1.256	.231	0.495	−0.195	1.186	.160
Maternal care	0.084	−0.211	1.891	.117	0.954	0.023	1.885	.045
Mental health care	0.255	−0.701	1.211	.601	0.524	0.023	1.885	.225
Pediatric care	1.020	0.180	1.859	.017	0.899	0.155	1.642	.018
Nondirect care	1.047	0.117	1.978	.027	1.772	0.948	2.597	<.001
*F* value	35.42 (13, 690)			<.001	5.31 (13, 690)			<.001
Adjusted *r*^2^	.39				.07			

*Note. N* = 704. The total score of Families’ Importance in Nursing Care—Nurses’ Attitudes was used as the dependent variable. Nonsignificant interactions between country and predictors were removed. Fam-RNC = family as a resource in nursing care; Fam-CP = family as a conversational partner; Fam-B = family as a burden (reverse scores); Fam-OR = family as its own resource; CI = confidence interval.

a Country reference group = Sweden. ^b^ Education reference group = first obligatory nursing diploma or degree. ^c^ Gender reference group = female. ^d^ Have had a seriously ill family member in need of professional care. ^e^ Practice area reference group = medical-surgical unit.

The models of the predictors of each of the subscales are found in Table 4. Of the four subscales, the predictors accounted for most variation in the Fam-B subscale (39%) and least variation in the Fam-OR subscale (7%). Age was a significant predictor across all four subscales (outcomes; *p* < .001), while country was only associated with scores on the Fam-B subscale. Once all model variables were considered, attitudes of family as more of a burden (lower scores) were found in Hong Kong, China (*B* = −4.6, 95% CI = [−5.2, −4.1], *p* < .001), and Ontario, Canada (*B* = −1.8, 95% CI = [−2.4, −1.3], *p* < .001), compared with Sweden.

Practice area was associated with each of the subscales, but the pattern of association varied. In comparison with medical-surgical areas, nurses working in geriatric care were associated with higher scores in the Fam-RNC and Fam-CP subscales. Working in maternal care was also related to higher scores in the Fam-RNC and Fam-CP subscales, as well as higher scores in the Fam-OR subscale. Working in mental health care was associated with higher scores in the Fam-CP subscale, while working in pediatrics was associated with higher Fam-B scores (perceived family as less of a burden), as well as higher scores in the Fam-CP and Fam-OR subscales. By contrast, working in critical care was associated with lower scores (less positive attitudes) in the Fam-B subscale and lower scores in the Fam-RNC subscale. Working in nondirect care was consistently associated with more positive attitudes in all subscales. Finally, being male was associated with lower scores in the Fam-CP subscale (*B* = −1.8, 95% CI = [−3.1, −0.6], *p* = .003). Education level and having had a seriously ill family member in need of professional care were not significantly associated with any of the outcomes examined.

### Objective 2b. Differences in Predictors by Country

The lack of evidence supporting the presence of statistical interaction suggests that the effects of the predictors on nurses’ attitudes were similar across the countries tested.

## Discussion

To our knowledge, this is one of the first studies to examine nurses’ attitudes about the importance of family involvement in nursing care across all health care settings from an international perspective. Our study objectives were to describe and compare the level of nurse attitudes of the importance of family in nursing care across three countries; to identify predictors of nurse attitudes toward family importance in nursing care; and to determine whether predictors vary by country. We found that country, age, gender, and practice area were significant predictors, and that all model predictors accounted for 18% of the total variation in nurses’ overall attitudes (total scores) toward the importance of family in nursing care. In the model of Fam-B, the predictors accounted for nearly 40% of the variation, a much higher percentage than that accounted for in the overall scale or the other subscales. Significant predictors of family as a burden included country, age, and practice areas. Examining the standardized regression coefficients, the effect of working in Hong Kong, China, compared with Sweden was much larger relative to the other model effects with family as a burden as the outcome; however, with the other subscales as outcomes, the relative difference in effects was much smaller. A similar pattern was also found for the effect of working in Ontario, Canada, compared with Sweden. By contrast, the effect size of age and practice area relative to the other model effects was more consistent across all the models of the subscales. This suggests that the effect of country is more apparent in relation to attitudes of family as a burden, highlighting there may be cross-country differences in health care/workplace policies regarding the presence of family and their involvement in care.

Nurses working in Hong Kong, China, had significantly less positive attitudes toward the importance of family in nursing care than nurses working in Ontario, Canada, or Sweden. We located only one other study that compared nurse attitudes across several countries in their sample (Luttik et al., 2017). Luttik and colleagues (2017) found that nurses working in Scandinavian countries (i.e., Denmark, Norway, Sweden) had more positive attitudes than nurses in Belgium. As noted, there may be differences between countries in family involvement in nursing care (Luttik et al., 2017).

While the proportion of male respondents was low overall, and particularly in Ontario, Canada, we found that gender had an effect on the overall scores. This was largely derived from the Fam-CP subscale, where men were found to have less positive attitudes about family as a conversational partner than women. Studies conducted in Sweden similarly found that men had less supportive attitudes for family as a conversational partner (Benzein, Johansson, Arestedt, & Saveman, 2008; Linnarsson et al., 2014). Gender differences where men had less positive attitudes than women have also been reported for the subscales Fam-RNC (Benzein, Johansson, Arestedt, & Saveman, 2008) and Fam-OR (Linnarsson et al., 2014; Sveinbjarnardottir et al., 2011). While it is not clear why these gender differences exist, these findings may be due to cultural differences between countries, or differences between male and female communication styles. In a review of studies examining gender differences in health care provider-patient communication in medical encounters, Street (2002) noted that research has suggested that men and women tend to have different communication styles, which is associated with one’s socialization (e.g., gender roles, cultural norms, values, beliefs, attitudes). For example, female health care providers may be more interpersonally and relationally oriented such as building partnerships with patients than male health care providers (Street, 2002). In a recent study exploring male nurses’ views of gender in the nurse-family relationship in pediatric care, male nurses described how they exerted more control over the boundaries of relationships with families including limiting their emotional involvement than their female colleagues (Arreciado Marañón et al., 2019). Street (2002) highlighted that one’s attitudes toward men and women may generate assumptions or gender-based beliefs about the capabilities and needs of conversational partners. However, other studies found no association between gender and nurses’ attitudes toward the importance of family involvement in nursing care (Alguire, 2013; Hoplock et al., 2019; Luttik et al., 2007). Ethnicity, age, and other factors including the broader context of health care (e.g., political, cultural) may also influence communication patterns and interactions (Street, 2002).

Practice area was a significant predictor of nurses’ attitudes. On average, nurses working in critical care had significantly less positive attitudes about the importance of family in nursing care overall compared with those working in medical-surgical areas. Those in practice areas of geriatric care (e.g., long-term care), maternal care, pediatrics, and nondirect care had significantly more positive attitudes compared with the medical-surgical practice area. Practice areas were also associated with each of the subscales. For example, in comparison with nurses working in medical-surgical units, nurses working in maternal care had more positive attitudes about family as a resource in nursing care, family as its own resource, and family as a conversational partner. Nurses working in geriatric care, pediatrics, and mental health also reported more positive attitudes toward family as a conversational partner. Nurses working in pediatrics and in nondirect care roles perceived families as less of a burden. Previous studies examining various specializations or work settings have reported differences in nurse attitudes. For example, intervention studies conducted in Iceland have reported variation in nurse attitudes between different psychiatric units (Sveinbjarnardottir et al., 2011), and between outpatient and day surgery departments and inpatient departments (Blondal et al., 2014).

Studies comparing hospital settings with home care or primary health care have been mixed. Researchers have found that nurses working in home care (Hagedoorn et al., 2020) or primary health care (Benzein, Johansson, Arestedt, & Saveman, 2008; Gusdal et al., 2017; Hagedoorn et al., 2020; Østergaard et al., 2020) reported more positive attitudes than those working in hospitals. However, Hoplock et al. (2019) reported no differences in nurses’ attitudes among hospital and home care settings. In this study, there were no significant differences in attitudes toward the importance of family in nursing care between nurses working in primary care/home/community care compared with those working in medical-surgical areas.

Nurses working in nondirect care roles reported more positive attitudes across all four subscales. Nurses in these roles may experience family involvement in care differently. This finding is consistent with other studies that have reported more positive attitudes among researchers (Luttik et al., 2017), educators, and managers (Alguire, 2013; Luttik et al., 2017). As noted by Alguire (2013), nurses in roles such as a manager or educator tend to spend less time at the bedside, which limits their exposure to families and may explain more positive attitudes. Luttik and colleagues (2017) further noted that it can be difficult to implement a family-focused approach in clinical practice, particularly when there are time constraints or a lack of experience with involving families in care (Benzein, Johansson, Arestedt, & Saveman, 2008). Actively involving family in care requires support from the team including nurses, physicians, and other health care professionals (Liput et al., 2016). Studies examining health care professionals’ attitudes toward the involvement of family in critical care and pediatric care have found that health care professionals have positive attitudes toward family involvement in routine care, but they had less supportive attitudes toward family presence during resuscitation efforts in critical care (Al Mutair et al., 2014) or complex, technical tasks in the care of hospitalized children (Power & Franck, 2008). Studies examining health care professionals’ attitudes toward family involvement in care have focused on specific care situations such as family presence during resuscitation and other invasive procedures (Al Mutair et al., 2014). Liput and colleagues (2016) conducted a literature review that explored both health care professionals and family attitudes toward involvement in intensive care and found that they share an attitude that a partnership is essential to provide optimal care. Strategies are needed such as education and training programs to facilitate family integration into the model of care (Al Mutair et al., 2014; Liput et al., 2016).

In this study, the education level was not a significant predictor of nurses’ attitudes toward family importance in nursing care. While this finding is consistent with previous research (Hoplock et al., 2019; Linnarsson et al., 2014), other studies reported an association between higher education level and more positive attitudes (Gusdal et al., 2017; Hagedoorn et al., 2020; Luttik et al., 2017; Østergaard et al., 2020; Sveinbjarnardottir et al., 2011). However, we assessed education based on the first professional training requirement in nursing (diploma or bachelor’s degree) and a higher level of postgraduate education (e.g., master’s, PhD) and the requirements for basic licensure varied across countries.

Studies examining the impact of an education or training intervention for nurses on the importance of involving family in care have shown that nurses perceived families as less burdensome following training (Sveinbjarnardottir et al., 2011; Yamazaki et al., 2017), and nurses’ understanding of the importance of family in care was strengthened (Yamazaki et al., 2017). While Blondal et al. (2014) reported no differences in nurses’ attitudes before and after their educational intervention, they suggested tailoring interventions to practice areas. Including a control group in intervention studies may also be warranted. Interventions such as education or training that are tailored to the practice area, and aim to develop skills and competencies in communicating and collaborating with families as active partners in the care process, are approaches that could support family-focused care (Benzein, Johansson, Arestedt, & Saveman, 2008; Hoplock et al., 2019; Hsiao & Tsai, 2015; Linnarsson et al., 2014; Luttik et al., 2017; Østergaard et al., 2020). For example, the International Family Nursing Association (IFNA, 2015) outlined nurse competencies for generalist family nursing practice centered around five core competencies:

(1) enhance and promote family health; (2) focus family nursing practice on families’ strengths/ the support of family and individual growth/ the improvement of self-management abilities/ the facilitation of successful life transitions/ the improvement and management of health/ the moblilzation of family resources; (3) demonstrate leadership and systems thinking skills to ensure the quality of nursing care with families in everyday practice and across every context; (4) commit to self-reflective practice with families; and (5) practice using an evidence-based approach. (p. 3)

Mentorship programs for novice nurses and manager support for allocating dedicated time for nurses to establish trusting relationships with patients and family members could also contribute to meaningful family involvement in nursing care (Benzein, Johansson, Arestedt, & Saveman, 2008; Gusdal et al., 2017; Hsiao & Tsai, 2015). Other modifiable factors of the work environment such as ensuring that best practice guidelines, policies (e.g., visitor policies), and workplace philosophies are in place that encourage family involvement in care are additional strategies that may support family involvement in care (Hoplock et al., 2019).

Providing client- and family-centered care is an entry-level competency for registered nurses in the three countries included in our sample (College of Nurses of Ontario, 2018; The Nursing Council of Hong Kong, 2012; Swedish Nurses’ Association, 2017). However, the content and amount of training in family-centered care varies across these nursing programs, and family-centered care may not be well integrated into all practice areas (Hsiao & Tsai, 2015). Gaining an understanding about differences between countries in regard to attitudes toward family nursing has implications for both practice (e.g., learning from other countries’ health care systems, policies) and education (e.g., understanding how nurses are trained to fulfill the core competencies outlined by the IFNA, 2015).

Future research should seek perspectives from various stakeholders such as registered/licensed practical nurses, nurse practitioners, other health care professionals, and families for a more comprehensive understanding of attitudes and factors that may contribute to family involvement in nursing care (Blondal et al., 2014; Hoplock et al., 2019). Research should further explore nurse attitudes toward family importance in care between direct clinical practice roles and nondirect nursing roles. Aside from the study conducted in Belgium and Scandinavian countries by Luttik and colleagues (2017), we located no other studies that examined nurse attitudes of family importance in care from an international perspective. Research should further explore cross-country differences in nurse attitudes, as well as the role of culture in nurse attitudes toward family involvement in nursing care (Luttik et al., 2017). Knowing where cross-country differences occur could inform targeted interventions and provides areas of research for future international comparative studies. Qualitative studies exploring cultural or cross-country differences may provide additional insights.

To our knowledge, ours is the first study using the FINC-NA to examine nurse attitudes toward family importance in care that has included long-term care settings. Research that examines nurse attitudes toward family involvement in care in long-term care settings could inform targeted interventions in this setting. New and innovative ways to involve families in care should be explored.

### Limitations

Our study provides a comprehensive understanding of nurse attitudes toward the importance of family in nursing care from across all health care sectors in Hong Kong, China, Ontario, Canada, and Sweden. However, there are limitations to note. We used a convenience sample including snowball sampling which may affect results; for example, people are connected by social media to people who tend to hold the same views. While accessing potential participants through online social media can be a feasible and effective recruitment strategy (Whitaker et al., 2017), only nurses with access to online nursing professional groups and other online nursing interest groups or social media groups had access to the survey. However, online data collection allows participants to complete the survey at their convenience, and data are anonymous. While participants were invited to complete the survey only one time, we cannot ensure that participants completed the survey only once. The use of representative samples in future research will be important to validate our model findings and demonstrate generalizability to wider nursing populations.

Sample sizes varied across countries, and in two of the three countries, the target sample size was not met despite the use of best practices of internet recruitment and a long recruitment period. Our data collection period and participant recruitment coincided with social unrest in Hong Kong, China, and it overlapped with the onset of COVID-19 in spring 2020. As the statistical power of a comparison was determined by the smallest sample size, cross-country comparisons with Ontario, Canada, had less power to detect a difference. In addition, as sample sizes to test interactions are smaller than those for main effects, the power to detect interactions was also limited due to smaller sample sizes in Ontario, Canada, and Hong Kong, China, compared with Sweden. Future research should obtain samples across countries that are large and similar in size. Ideally, more countries would be included so that between-country variation would be better understood using multilevel models. In this study, we could not determine the nature of the cross-country differences. For example, these could reflect different health care policy environments or differences in cultural values. A recent qualitative study reported that factors such as the organizational environment, the patient’s condition, and the nurse’s attitudes and perceptions of family were factors that resulted in variation in practices for involving families in care in intensive care units (Naef et al., 2021). However, the manner in which nurses involve families in care is not well understood (Misto, 2018; Naef et al., 2021). Future studies making cross-country comparisons should explore the cultural, relational, and organizational context (e.g., organizational policies for family involvement in care, guidelines) with regard to nurses’ attitudes and the role of the family in nursing care (Naef et al., 2021).

We only surveyed nurses in Ontario, Canada. While Ontario is the most populous province in Canada, the sample size was low and it provides only a snapshot of one province within Canada. There were a low number of males included in the study, particularly in Ontario, Canada. This would have reduced the power to detect gender effects, but also interactions between gender and country. Future research should focus on obtaining a larger sample of male nurses to better understand the effect of gender, and use a qualitative approach to explain gender differences in attitudes toward the importance of family in nursing care.

The internal consistency for the Fam-B subscale for Hong Kong, China, was lower (α = .62) than that for the other two countries, suggesting that some items may be heterogeneous. Previous studies have reported a Cronbach’s alpha of <.70 for the Fam-B subscale (Benzein, Johansson, Arestedt, & Saveman, 2008; Blondal et al., 2014; Linnarsson et al., 2014). As Blondal et al. (2014) noted, because this subscale contains fewer items (four items), a Cronbach’s alpha of .60 or greater is acceptable (Nunnally & Bernstein, 1994). The FINC-NA has not been previously tested in Hong Kong, China. Moreover, the FINC-NA has not, to our knowledge, been tested for measurement invariance across languages; therefore, comparison of the mean scores needs to be done with some caution. Future international work using this instrument should be preceded by formal testing of measurement invariance across languages to provide stronger evidence of cross-national differences. In addition, we developed an education variable for which the requirements for basic licensure varied across countries. Finally, categories of practice areas were developed in an effort to be consistent and these may not have corresponded exactly across the three countries. However, including nurses working in a variety of practice areas across health care settings may increase the generalizability of our study findings.

## Conclusion

Results from this study indicated that country, age, gender, and practice areas were factors that were associated with nurses’ attitudes toward the importance of family in nursing care. On average, nurses working in Hong Kong, China, had less positive attitudes compared with Ontario, Canada, and Sweden, with most of the difference in Hong Kong accounted for by stronger perceptions of family as a burden. Our study advances knowledge of nurses’ attitudes toward the importance of family involvement in nursing care from a multinational perspective. Findings could lead to the development of education programs or interventions that tailor the nursing care offered to families. The identification of cross-national differences signals the need to investigate the role of culture and health care system-level factors that may contribute to nurses’ attitudes toward the importance of family in nursing care.

## Supplemental Material

sj-docx-1-jfn-10.1177_10748407211042338 – Supplemental material for Nurses’ Attitudes Toward the Importance of Families in Nursing Care: A Multinational Comparative Study Click here for additional data file. Supplemental material, sj-docx-1-jfn-10.1177_10748407211042338 for Nurses’ Attitudes Toward the Importance of Families in Nursing Care: A Multinational Comparative Study by Lisa A. Cranley, Simon Ching Lam, Sarah Brennenstuhl, Zarina Nahar Kabir, Anne-Marie Boström, Angela Yee Man Leung and Hanne Konradsen in Journal of Family Nursing
